# The role of mediators in undergraduate field experiences: Suggesting additional perspectives to the UFERN model

**DOI:** 10.1093/biosci/biaf005

**Published:** 2025-04-23

**Authors:** Pascal Schaldach, Matthias Wilde

**Affiliations:** Department of Biology Education (Zoology & Humanbiology), Faculty of Biology, Bielefeld University, Bielefeld, Germany; Department of Biology Education (Zoology & Humanbiology), Faculty of Biology, Bielefeld University, Bielefeld, Germany

This correspondence is in response to the article “A tool for designing and studying student-centered undergraduate field experiences: The UFERN model,” by O'Connell and colleagues (2022), published in *BioScience* (https://doi.org/10.1093/biosci/biab112), which introduced the Undergraduate Field Experience Research Network (UFERN) model. The model provides a comprehensive framework for designing and evaluating inclusive, student-centered undergraduate field experiences (UFEs) by addressing student context factors, design factors, and student experiences that influence actual student outcomes. We commend the authors for developing this theory-based framework, which is a valuable tool for improving the quality of field education.

At Bielefeld University, we applied the UFERN model to field courses and found it to be an effective guide for structuring activities and assessing student learning outcomes. Its emphasis on understanding the interplay between individual student factors and course design elements has helped us create more intentional learning experiences. For instance, in one field course, we used the model to tailor activities to account for diverse student backgrounds, such as varying levels of prior knowledge and personal needs, which improved student engagement and learning outcomes. Our experience revealed a critical aspect not explicitly addressed in the original UFERN model: the pivotal role of the mediator (e.g., instructors, educators, or field leaders) in determining the success of UFEs.

In our courses, mediators were responsible not only for delivering content but also for dynamically adapting the design factors in response to real-time challenges. For example, during a course involving ecological sampling, unexpected environmental changes required the mediators to redesign planned activities on site, ensuring that the course objectives remained achievable. The mediators drew on their professional knowledge, self-regulation skills, and capacity for situational problem-solving—competencies that were crucial in maintaining the quality of the learning experience. Research supports the importance of mediator competencies, with studies highlighting the role of professional expertise, beliefs, and adaptability in enhancing educational outcomes (Shulman 1986, Helmke 2006, Hattie and Timperley 2007, Baumert and Kunter 2011, Hattie 2012, Biggs and Tang 2014, Vermote et al. 2020).

Furthermore, the mediators’ self-reflection emerged as a key factor influencing the improvement of the UFEs. For example, after each course, our mediators evaluated how their teaching methods, real-time decisions, and interactions with their students affected those students’ learning outcomes. These reflective practices led to iterative refinements in course design that made subsequent UFEs more effective. Prior research also underscores the link between mediator self-reflection and improved teaching quality (Korthagen and Kessels 1999, Hattie and Timperley 2007). This suggests that mediators’ ability to critically evaluate and adapt their practices plays a crucial role in shaping both student experience and actual student outcomes, but this dynamic is underrepresented in the UFERN model.

To address this gap, we propose an extended UFERN model that incorporates mediator factors as a component (see figure 1). Mediator factors should include professional knowledge (e.g., pedagogical and subject-specific expertise), beliefs and values, motivation, and adaptability. These factors influence the effectiveness of design factors and their alignment with student context factors, creating a feedback loop that enhances student experience and actual student outcomes. Mediators also act as bridges between the theoretical framework and its practical application, translating the model's design principles into actionable strategies that resonate with diverse student populations.

**Figure 1.**
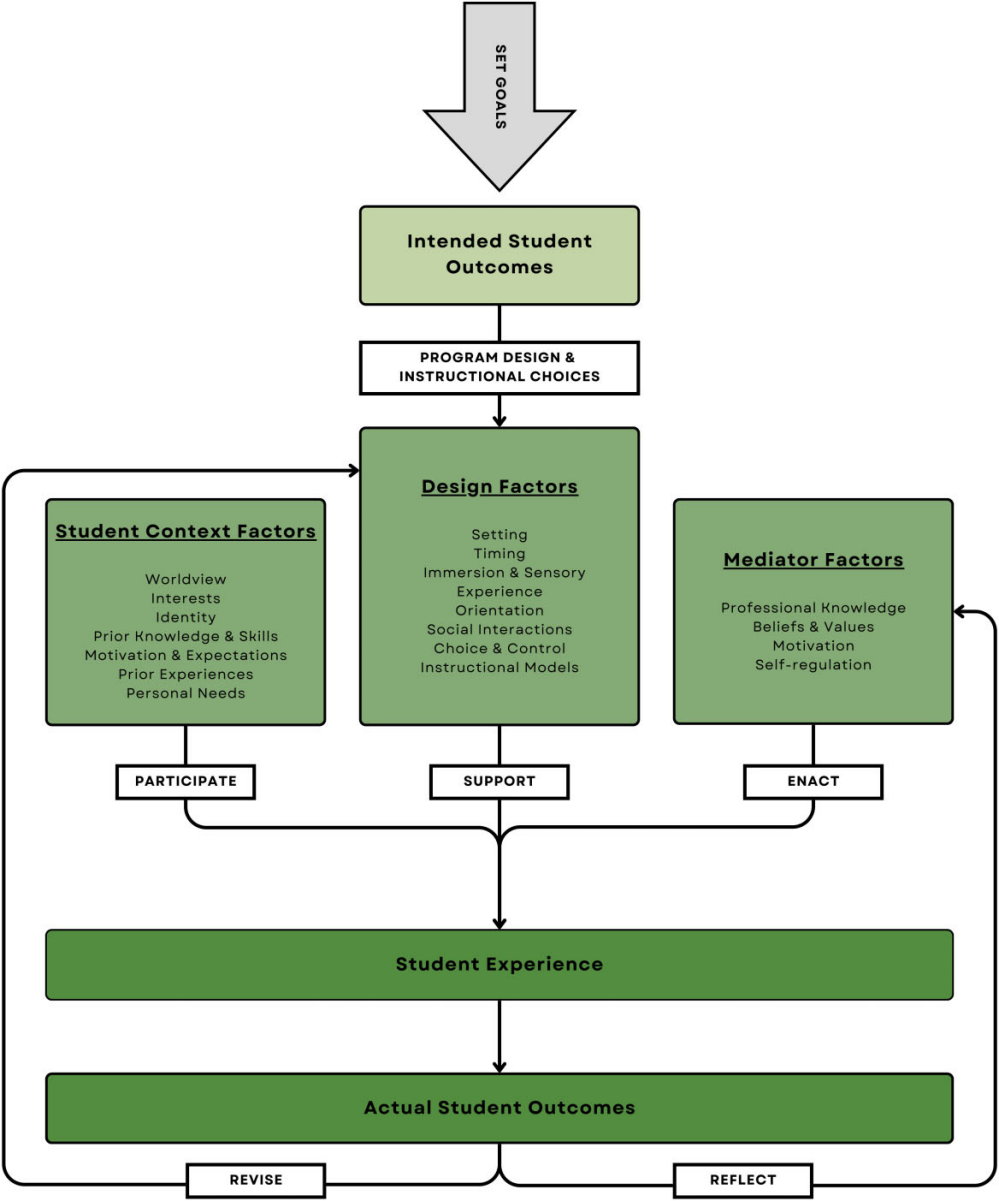
Adapted UFERN model.

Explicitly incorporating the mediator role might enhance the UFERN model's utility by recognizing mediators as active agents in the learning process. This addition could help educators develop competencies to address unforeseen challenges and foster inclusive, student-centered environments. Future empirical work could explore how mediator factors interact with other model components.

In conclusion, although the UFERN model is a valuable tool, integrating mediator factors might improve its practical application, offering guidance for creating adaptable and inclusive field experiences in higher education.

## Author Biography

Pascal Schaldach (pascal.schaldach@uni-bielefeld.de) is a PhD student and Matthias Wilde is a professor in the Department of Biology Education (Zoology & Humanbiology), in the Faculty of Biology at Bielefeld University, in Bielefeld, Germany.
